# Why Are Enrichment Practices in Zoos Difficult to Implement Effectively?

**DOI:** 10.3390/ani12050554

**Published:** 2022-02-23

**Authors:** Eileen K. Tuite, Simon A. Moss, Clive J. Phillips, Samantha J. Ward

**Affiliations:** 1 College of Health and Human Sciences, Charles Darwin University, Darwin, NT 0810, Australia; simon.moss@cdu.edu.au; 2 Institute of Veterinary Medicine and Animal Sciences, Estonian University of Life Sciences, Kreutzwaldi 1, 51014 Tartu, Estonia; clive.phillips@curtin.edu.au; 3 Curtin University Sustainability Policy Institute, Curtin University, Kent St., Bentley, WA 6102, Australia; 4 School of Animal Rural and Environmental Sciences, Nottingham Trent University, Southwell NG25 0AJ, UK; samantha.ward@ntu.ac.uk

**Keywords:** enrichment, big cats, keepers, zoo management, thematic analysis, process evaluation

## Abstract

**Simple Summary:**

Zookeepers often introduce enrichment practices in which they provide animals with diverse stimuli and challenges to stimulate mental and physical activity, but enrichment can be difficult to implement and the enrichment used in zoos is not always effective at improving animal welfare. Limited information is available on how zookeepers apply enrichment practices in zoos. To identify some of the reasons that zoos cannot always provide effective enrichment, we interviewed keepers across multiple zoos. Keepers that work with big cats were deemed particularly well suited to the study, given the extensive recommendations in the literature for this group of animals in zoos. We discovered that many impediments obstructed the capacity of zookeepers to enrich animals effectively, such as conflicting priorities, uncertainty about which enrichment practices are effective, and concerns about the perceptions of visitors. To illustrate, zoos often impose routines for keepers to address more immediate and visible problems, such as sick animals or visitors who do not like unnatural objects in the enclosure. Hence, the enrichment and stimulation of animals is not always perceived as important or pressing and, therefore, may be deferred or neglected. We suggest that greater clarity and increased transparency on the objectives and means of assessing enrichment could curb confusion surrounding enrichment practices in zoos.

**Abstract:**

The good intentions of zoos to introduce enrichment practices that stimulate animals mentally and physically are not always achievable. Changes to the policies and procedures in organisations are difficult to fulfil for a range of reasons frequently investigated in change management literature. The implementation of these changes can be the source of ineffective attempts to generate positive interventions in organisations. In this study, we investigate whether interventions to improve animal management in zoos through enrichment are subject to implementation impediments. Qualitative data gathered from interviews with 23 keepers working with big cats across 12 zoos globally provided valuable insights into the barriers and enablers to the implementation of enrichment. Keepers participated voluntarily and worked in accredited zoos across Australia, New Zealand, Europe, south-east Asia, South Africa, and the United States of America. Thematic analysis of the data revealed five key themes that described some of the challenges zoos and keepers experience when implementing enrichment for big cats, in their words: *“let’s just be cautious”*, *“purely surviving”*, *“struggle to understand the goal”*, *“can’t always provide what you should”*, *and “judge the effectiveness”*. These themes provide additional insights into potential areas for improvement, including greater attention to the benefits of enrichment for animal mental health and increased transparency around enrichment objectives in zoos.

## 1. Introduction

Organisations often have the objective to change policies, procedures, and practices to improve productivity and performance. Yet, these aspirational intentions of most organisations are seldom fulfilled [1]. To address these challenges, researchers have explored a wide range of reasons that organisational change efforts are unsuccessful.

Changes to organisational operations are often unsuccessful because plans to improve practices and procedures are not implemented effectively. To understand the source of these failures, some researchers closely investigate specific interventions in organisations to explore how programmes are implemented and operate in practice [2]. This research approach, often called Process Evaluation, is commonly adopted in the health and education sectors to investigate the range of processes that hinder the success of interventions [3,4,5].

Investigations into the processes that underpin implementation often reveal details that are not otherwise evident from evaluating outcomes alone [6]. To illustrate, one study applied Process Evaluation to investigate an intervention that was designed to improve public health. This programme had not generated the anticipated health outcomes. The study identified that only some, but not all, the stages of implementation were delivered as intended [7]. This approach, therefore, can guide decisions on how to improve programmes [8], increasing the likelihood that desired outcomes are achieved. Conversely, some interventions may appear to be implemented as intended but still fail to achieve the desired outcomes [9], suggesting the underlying theory of change or assumptions may need to be modified.

### 1.1. Enrichment Practices in Zoos

One instance in which a programme may not always be implemented as intended revolves around enrichment practices in zoos. Zoos introduce enrichment to improve the management and welfare of animals [10]. Problems with the implementation of enrichment may, however, hinder the overall effectiveness of these practices.

To implement enrichment practices, zookeepers (hereon in keepers) introduce novel and diverse sensory stimuli, such as objects, materials, and training practices that engage animals in active behaviour [10,11]. Enrichment practices are designed to elicit a diverse repertoire of physical behaviours, stimulating and fulfilling the cognitive [12], social [13], and emotional needs of animals [14]. Such enrichment routines, when planned carefully, varied regularly, and assessed reliably, can appreciably enhance the welfare of captive animals [15].

Enrichment practices that are not delivered as intended may not always fulfil the cognitive, social, and emotional needs of animals. As research indicates, enrichment can elicit behaviours that are typical of a range of species in nature and diminish particular signs of problems—that is, abnormal behaviours such as pacing and other stereotypies (for reviews see [16,17]). However, the enrichment practices of zoos do not always achieve the intended outcomes [18]. Indeed, studies indicate that keepers cannot always implement enrichment practices as planned. For example, keepers are sometimes too occupied with other duties to contemplate enrichment [19] or they may not have developed the capacity to select appropriate enrichment that is beneficial for the animals [20]. Yet, despite these examples, evidence on whether enrichment practices are implemented as intended is sparse [17].

Few studies have investigated the perceptions of people that shape enrichment practices. There is evidence that zoo staff, when reaching decisions about enrichment and other practices, consider the perceptions of visitors towards the animals, exhibits, and the institutions [21,22,23,24] and not merely the welfare of animals [25]. However, studies are yet to investigate how visitor perceptions shape the development of enrichment protocols and decision making in zoos.

### 1.2. Social Sciences in Zoo Research

Interest in the perspectives of keepers is evident in an increase in qualitative investigations in zoos. Studies in zoos on both people and animals have hitherto largely relied upon quantitative data gathering and analysis (for example [26]). More recent trends to gather broader insights into the experiences of zoo professionals and practitioners is evident in the uptake of qualitative research approaches [27,28,29]. For example, research into bonds between keepers and animals has predominantly relied on quantitative investigations [30] but more recently, studies of this topic have incorporated qualitative approaches to yield deeper insights to inform future animal management in zoos [27].

To gain a more complex understanding of enrichment practices in zoos, and to explore some of the tensions, contradictions, and paradoxes that keepers need to reconcile, advancements beyond traditional hypothesis-driven studies can be facilitated by qualitative research approaches [17,31]. One such study incorporated both qualitative and quantitative analyses of the opinions of zoo professionals on the application of enrichment across different species [32]. These studies contributed to our broader knowledge of enrichment perceptions but are still largely driven by a deductive approach to address targeted research questions.

Investigations that apply an inductive form of inquiry through qualitative research methods are less common in the zoo setting, with only one account found to date [33]. This account provides a unique analysis of both the personal and professional nature of the keeper role, highlighting ways in which keepers assign meaning to their work. Studies that apply this exploratory approach are particularly well suited to better understand keepers, and people more generally, to make sense of how individuals generate beliefs and interpret their own behaviours, experiences, and interactions [34].

Qualitative research provides the foundations of inductive inquiry investigations more prevalent in the social sciences. Such research approaches—often designed to understand rather than predict—apply open-ended or exploratory investigation methods. Qualitative investigations have been applied in the health industry [35] to better understand practices through the opinions and perspectives of practitioners [36]. Studies that rely on this inductive, rather than deductive, approach reveal nuanced insights into the experiences of nurses, patients, and doctors [37].

### 1.3. Present Study

Consistent with a qualitative research approach, the present study was designed to explore the reasons that enrichment practices in zoos are not always effective in achieving the intended outcomes. To do so, we adopt a perspective of social constructionism to investigate and characterise the perceptions, beliefs, attitudes, interactions, and experiences of keepers who implement enrichment. Research informed by social constructionism recognises the subjective experiences of people and acknowledges the role of societal and cultural interactions in shaping the perspectives of individuals [38,39]. Thus, keepers in the present study were not assumed to be independent entities responsible only for husbandry and enrichment practices, but rather active participants in the social environment of both the zoo and human society.

In the current study, we attempted to clarify the barriers that impede the implementation of enrichment and the enablers that facilitate enrichment practices. First, rather than focus on the objective outcomes of enrichment, we explored the processes and procedures of enrichment implementation that are both effective and ineffective through a Process Evaluation framework. Second, we incorporated an analytical framework known as Actor Network Theory to investigate the networks of people, animals, and objects involved in both the application of enrichment practices and outcomes.

Specifically, enrichment often involves complex processes and interactions between individuals responsible for developing and implementing enrichment in zoos. To explore this complexity, this study applied certain principles from the Process Evaluation (PE) framework [2]. PE offers a comprehensive framework that directs the attention of researchers to the mechanisms that translate the implementation practices to the various outcomes and to how the context tempers or amplifies these mechanisms [2]. Researchers apply PE to explore the experiences and social interactions of practitioners and to uncover previously unexpected details of whether practices were delivered as intended [40]. Furthermore, the PE framework also underscores how the outcomes and contexts affect the behaviour of people who enact the interventions [3,41], such as the keepers.

In addition to this Process Evaluation framework, another perspective, Actor Network Theory (ANT) [42,43], also guided our interpretation of results. Specifically, enrichment practices consist of an interplay between animals in captivity, objects, such as enrichment items, policies, guidelines, and people, such as keepers and managers. In this instance, the animals, objects, and people are important actors and through their actions, forming dynamic chains of association [44]. ANT sensitises researchers to the reciprocal interaction between different actors and provides a useful lens to consider the complex nature of relationships between humans and animals [45]. That is, Actor Network Theory encourages researchers to contemplate how the diverse capabilities of humans and non-humans influence one another to culminate in various consequences [46,47]. For example, the configuration of enclosures—an object—can affect which enrichment items the keepers may choose and ultimately the behaviour of animals. The behaviour of animals can then guide other changes to the enclosure [48] and so forth.

In the current study, we sought to explore conditions that affect the capacity of keepers to implement enrichment effectively. We applied a qualitative approach, in which we interviewed keepers and subjected their responses to a thematic analysis [49]. Throughout the project, we refined the interview questions, the coding schemes, and the ensuing themes iteratively [50].

## 2. Materials and Methods

Inspired by more recent trends in the field [27,29], we used an exploratory and qualitative research method that diverges from a previous emphasis on quantitative methods to investigate enrichment implementation in zoos. Specifically, we relied on an inductive approach to explore and uncover new insights, unencumbered by a priori assumptions more common in hypothesis-driven investigations [51]. Consistent with this exploratory approach, we collected data relevant to the topic to understand the experiences and perceptions of keepers who implement enrichment for big cats. Thus, our study was intended to uncover concepts that may or may not already be discussed in the research literature.

The current study also forms part of a larger body of work into the practices, policies, and procedures that could better foster enrichment in zoos. Thus, a subset of data from this area of investigation was analysed to explore the specific research topic outlined.

### 2.1. Data Collection

#### 2.1.1. Study Sites

Under the direction of purposeful sampling, we invited keepers and head keepers to participate from selected zoos that met specific criteria. Purposeful sampling is intended to provide meaningful information that is relevant to the purpose of the inquiry [51]. To sample participants for the current study in this manner, we selected keepers using two criteria. First, we invited both keepers and head keepers who currently or previously worked with big cats. Second, the keepers volunteering to participate needed to work or have worked in a zoo with institutional accreditation.

In the current study, we targeted one group of animals—big cats—to characterise in greater depth the complexity of barriers and determinants of enrichment implementation across several zoos. We considered the term big cats to broadly describe animals in the family Felidae, including tigers, lions, leopards, and cheetahs. Several species of big cats have been documented to be particularly hard to accommodate [52,53] and are inclined to exhibit signs of impaired welfare unless enrichment is suitable [54]. Consequently, a study that is confined to big cats should unearth a range of meaningful insights into the challenges of implementing enrichment practices as well as strategies to address these challenges. Big cats are also well represented in many zoos globally.

Zoos without accreditation were excluded, partly because welfare standards might not be adequate, posing an ethical conflict for the research as well as including accounts of inappropriate enrichment practices that are less likely to apply to accredited zoos. Each zoo was listed as a member of the World Association of Zoos and Aquariums (WAZA), a global alliance, with each zoo accredited by their respective regional zoo association. The zoo accreditation bodies of each region include American Zoo and Aquarium Association; Zoo Aquarium Association of Australasia; Southeast Asian Zoos and Aquariums Association; European Association of Zoos and Aquaria; British Association of Zoos and Aquariums; and Pan-African Association of Zoological Gardens and Aquaria.

#### 2.1.2. Data Gathering

Data were derived from semi-structured in-depth interviews, because this method enabled the interviewer to explore unanticipated insights about the research topic. Semi-structured interviews, common in the field of qualitative research, enable researchers to consider the perspectives and experiences of participants [34]. This open-ended interview approach encourages more reflexive discourse between interviewer and interviewee, rather than simply an extraction of information that could otherwise be collected through questionnaires [55]. Thus, interviews, and the research design more broadly, facilitated the exploration of emerging ideas in a flexible and adaptive manner [56].

Interview protocols followed a broad schedule of questions, explored within the categories of general keeper background, animal welfare concepts, enrichment practices, and overall enrichment development. The format of questions in semi-structured interviews for qualitative research rarely involve repeated series of pre-determined questions more common of the structured interviews in quantitative research [57]. Thus, many of the questions asked during each interview were adapted and modified iteratively as particular concepts became more salient [58]. In addition to scheduled questions, specific questions were also generated in response to participant comments [56]. To explore participant responses in greater depth, as well as to clarify responses, the interviewer—the first author, E.K.T.—asked non-directive probes and summarised previous responses, as suggested by Sarantakos [59], and illustrated in Table 1.

To accommodate the geographical distances and costs associated with sampling zoos globally, interviews were conducted remotely, both online and over the telephone. Telephone interviews [60] enabled greater flexibility to suit the schedule and preferred location of participants. These conditions also sometimes facilitate an increased sense of anonymity, enabling participants to contribute with greater openness [59].

#### 2.1.3. Study Participants and Ethics

To recruit participants, we needed to organise approvals from zoos—often by completing an official application—and specific keepers within these zoos. The first author, E.K.T., utilised established connections and professional networking events to recruit keepers. Keepers and head keepers who volunteered to participate were invited to take part in interviews once approvals were granted.

Sampling included more than one participant from each zoo where possible, using both opportunistic and snowball sampling. Snowball sampling involved asking participants whether other keepers might be interested in contributing to this study [61].

A targeted sample size of at least 10 is sufficient for semi-structured interviews that yield rich data [51,62]. Interviews were anticipated to last 45–60 min, with a minimum target sample size of 12 to ensure the data were sufficiently rich and nuanced [63].

The Human Research Ethics Committee at Charles Darwin University approved the proposed research and the proposed official interview schedule (Approval code H17129). All participants received a copy of the Plain Language Statement outlining details of the study, along with a consent form. Participants were invited to be interviewed at their convenience once consent forms were signed and returned to the first author, E.K.T. Invited participants were not obliged to participate and could choose to withdraw from the study at any stage before or during the interview process.

All interviews were audio recorded after participants signed consent forms and verbally consented before each interview. Audio recordings were saved and stored safely in accordance with Charles Darwin University research data management procedures. The data were initially identifiable, but then de-identified after transcription was completed.

### 2.2. Data Analysis

The first author conducted thematic analysis, adopting an inductive approach to diminish the biasing effect of preconceptions [49]. She completed the six phases that Braun and Clarke delineated [49], ensuring the approach was deliberative, reflective, and thorough.

During the first two phases, in which E.K.T. familiarised herself with the data, developed initial codes, and considered possible themes around the impediments and enablers of enrichment, E.K.T. also maintained a mindset in which she was receptive to new and unfamiliar concepts. In Phase 3, she assigned similar codes or concepts into clusters that represent potential themes. The analysis prioritised codes and themes that were relevant to the research questions rather than codes and themes that were merely frequent [49].

During the subsequent three phases, the initial themes were reviewed and refined. Comparison between and within initial themes enabled E.K.T. to interpret their implicit meaning, generating more abstract perspectives [49]. Thus, across the iterations, all the initial themes became increasingly encompassing, multifaceted, and nuanced. We developed themes that connect the specific properties of the environment in line with ANT [46]. Final themes were defined and named based on in vivo responses from participants. An illustration of these review stages—including initial and focused coding, early theme development, and final themes—appears in Table 2.

To generate the report, for the sixth and final phase, we compared the themes to the key principles of Process Evaluation, specifically the three properties of fidelity, dose, and reach. Fidelity represents the degree to which the practitioner observes the recommended standards or protocols of this intervention; dose represents the amount or magnitude of this intervention; and reach represents the extent to which the intervention is applied to a broad range of settings or circumstances [2].

Braun and Clarke [49] emphasise the importance of researcher positionality when applying thematic analysis. The first author, E.K.T., was the primary researcher responsible for gathering and analysing the data. During all phases of the study, E.K.T. adopted a reflexive approach, in which she monitored how her personal experiences and preferences may be biasing her interpretation of the findings [59].

## 3. Results

Keepers working in the carnivore department across 12 different accredited zoo institutions volunteered to participate. The current study was limited to keepers working with big cats. In total, 23 participants, including 19 keepers and 4 head keepers, were interviewed. Our findings are presented within the following description of five key themes, each consisting of two subthemes.

### 3.1. Let’s Just Be Cautious

Zoos adopt a largely risk aversive approach to enrichment procedures. This tendency to avoid both actual and perceived risks was manifested in both the enrichment practices applied by keepers as well as the culture of the zoo keeping community.

#### 3.1.1. “Sometimes Things Go Wrong”

To provide enrichment safely and responsibly, keepers often emphasised the dangers associated with enrichment practices. The potential risk of enrichment to the safety of animals or humans warrants constant monitoring and reassessing of certain items to minimise harm.

They gave the rhino a rubber tyre, and he got it around his head, and he almost died… it’s nice to give animals tyres … but sometimes things go wrong.

(ID15)

However, this vigilance appears to be somewhat exaggerated by fear and uncertainty, particularly surrounding the use of novel items. Consequently, a more widespread risk averse perception of enrichment appeared across most zoos.

Everything is going to kill an animal… I could have a boomer ball out there and the cat could hit it against the fence, and it could bounce back and do damage.

(ID34)

When providing enrichment, keepers often consider the risks not only to health and safety but also to the aesthetics. In particular, keepers express concerns for the appropriate presentation of enrichment given to animals on display.

[Management] are afraid of some guests complaining that the animals are playing with unnatural enrichment, responding to stuff they don’t find in the wild, and they don’t want to face these kinds of questions.

(ID21)

#### 3.1.2. “Don’t Think We Really Want the Attention”

Zoo managers attempt to regulate enrichment provisions to minimise unwanted or disparaging publicity. To reduce misunderstanding amongst visitors and the public, items that appear anomalous in the display or elicit unpleasant behaviours from animals were generally not permitted in many zoos. Consequently, various enrichment practices were limited.

It’s a tricky thing in a zoo, even more and more now, because there’s a lot of shift going towards a lot of natural environmental enrichment, not man-made items like treat balls you might give to your dog or cat at home.

(ID05)

The perceived risks of negative media attention and unfavourable public perceptions sometimes elicited comparable concern to the actual risks of unsafe practices for animals and people.

… [the vets] are just being cautious… we are in the spotlight so much… they don’t want to be on the front page saying *‘tiger died because vet said it was ok to give it alpaca wool*’.

(ID04)

This censoring of enrichment often limits the availability of desirable practices to enrich animals. Indeed, to avoid rhetoric against specific practices, zoos appeared to regulate what information keepers are permitted to share with the public. Thus, managers at zoos often limit the information that staff can disclose.

Emergency training—it was for any horror situation that we could be in from terrorism to animal activists, it covered all bases. And they said—look this does happen, people do come in and if they are asking questions that do feel very uncomfortable, then a keeper such as myself should call for some back-up ultimately and then they will assess the situation and have the right answers just in case I say the wrong thing.

(ID36)

Imposed limitations appeared to contravene a desire amongst keepers to communicate freely with visitors, the public, and the wider zoo community.

… you explain it to them and then they walk away going *‘wow, thank you, I’ve never known that*’ to me being a zoo, and being a keeper, we’re helping to educate the people

(ID07)

When zoos embody principles of transparency, keepers experience increased motivation to explore innovative approaches to enrichment collaboratively.

We all like to network and share resources amongst ourselves, because it’s really the only way to learn, there’s no point us trying to trail-blaze something if someone already has the knowledge.

(ID10)

Opportunities to share information with keepers from other institutes is particularly important for comparing and adapting enrichment practices on the successes and failures of past enrichment.

… we just have quite good connections with other zoos around us as well. So, when we do have issues or any questions or advice that we look for its very handy to be able to touch base with other collections and see how they are doing things.

(ID36)

However, across the industry more broadly, many zoos tended to obstruct transparency or they perpetuated a selective form of transparency that fulfilled specific objectives of the zoo. The same participant who described the benefits of inter-zoo connections also revealed that most zoos are unwilling to share their practices and experiences publicly. She attributed some of these limitations in transparency across zoos to the untrusting nature of the zoo community.

I think if one place starts to be a bit more open then hopefully it will lead to other collections being that way as well. But I think it would take a lot of time because it’s just so ingrained into us that you are not meant to show your weakness … I think slowly it is changing, it really is just a case of trust, I think as a community we are quite untrusting of each other.

(ID36)

Not all zoos discourage information sharing between institutes, but keepers in these zoos still describe limited opportunities for professional networking, often as a result of inadequate encouragement or support from their zoo.

… I wasn’t encouraged to network and seek other information and kind of learn more than what is just taught there.

(ID34)

Thus, selective transparency not only appeared common around discourse on enrichment protocols established by their institution but also extended into the culture of sharing in the zoo community.

I post a question; if it’s good enrichment and there is a discussion forum, but zoo people are more likely to discuss when the group is secret and put things in there.

(ID15)

### 3.2. Purely Surviving

Zoo managers often impose strict regulations on husbandry practices to fulfil the physical requirements of animals. Because of this emphasis, the policies, procedures, and practices of zoos often prioritise physical health and care over the mental health of animals. Consequently, keepers may not feel as obliged to foster mental stimulation in animals. Instead, keepers feel more compelled to avoid problems, such as animal malnutrition, than to promote positive animal experiences with enrichment.

#### 3.2.1. “Health and Wellbeing as Number One Priority”

Enrichment encompassed a range of practices that can be used to accommodate both the physical and mental health of animals. Yet, the fundamentals of animal husbandry were generally prioritised over enrichment, focusing on physical condition, hygiene, and reproductive success.

The zoo industry is very behind the eight-ball … I think it’s because historically, we have been all about cleanliness, as in high husbandry standards, and breeding animals.

(ID20)

Admittedly, some forms of enrichment also improve physical care, such as food provisions that address nutritional needs and facilitate exploratory behaviour or manipulation items that improve body condition and enable play.

You are trying to stimulate some kind of natural behaviour from them, so whether that be that you want to encourage play, or whether you want to encourage physical activity that would work say muscles they would if they were hunting.

(ID14)

However, enrichment practices that targeted mental stimulation of animals without the concomitant physical health benefits were not as likely to be encouraged by the zoo. Thus, enrichment intended to specifically address the behavioural, emotional, and cognitive needs of animals may not have received the same attention.

[The animals’] lives are good in terms of, having people to provide for them, taking care of them, medical attention. But there must be something more to it, not just this purely surviving, but their mental state of health is often disregarded.

(ID29)

Indeed, this prioritisation of physical care sometimes precluded certain enrichment practices; that is, these enrichment practices were specifically withdrawn or restricted to prevent damage to physical health.

… it smells like an animal, it’s part of an animal, it’s the fur. They’ve plucked it, they would play with it, they would roll in it. I guess the vets just look at it on a case-by-case basis and say there could be diseases that are passed on through that fur, so no we are not willing to allow that, we have to put our animals’ health and wellbeing as number one priority.

(ID04)

Physical care was also prioritised implicitly in the way staff at zoos monitor and enforce animal management practices. Zoo policies often delineated which procedures are essential to effective husbandry by outlining highly structured processes and routines. Standard operating procedures provide an example of zoo policy that effectively supported husbandry but could often impede enrichment practices.

Her den needs to be washed every Tuesday at 10 o’clock so at least I know it’s getting done once a week… so no, I think there are definitely benefits to having standards. But just locking-in times for these chores, particularly when it involves the provision of food and enrichment and training sessions, that’s the part where I would rather it be much more randomised.

(ID18)

Inadequate performance of husbandry procedures was often more likely to incur repercussions than not performing enrichment routines.

… if someone follows you the next day and the water bowl is empty you get reported, if you don’t put enrichment in you don’t get reported.

(ID09)

To avoid these admonishments, keepers often invested greater attention to animal husbandry. Limited accountability for enrichment reduced the efforts of many keepers, limiting the enrichment provided. Some keepers invested significant personal time and energy to enriching animals.

#### 3.2.2. “[Animals in Zoos] Would Still Survive without Enrichment”

This focus in zoos on husbandry duties over enrichment tends to encourage practices that are intended to avoid problems rather than create positive experiences for animals. Consequently, the attention of keepers was often directed to indicators that an animal is in decline, such as weight loss, fatigue, and abnormal behaviours.

I guess you’re looking for stereotypic behaviour or withdrawn behaviour that suggests something is awry that’s not letting them perform what they would do in a natural day.

(ID06)

Similarly, keepers often directed their attention more towards indicators of welfare deficit rather than noticing that an animal was content, playing, and behaving appropriately. Perhaps as a consequence, keepers sometimes regarded many enrichment practices that enhanced the lives of animals as subsidiary and optional.

It is definitely optional, because I’m pretty sure they would still survive without enrichment. But in terms of their welfare or how much fun they are having or whether their behaviour might change, it could definitely affect their behaviour in a positive manner.

(ID11)

Conversely, some keepers relied on enrichment to compensate for animal welfare problems rather than enhancing animal experiences. If an animal demonstrated some problem, such as pacing, keepers attempted to address this concern by introducing enriching changes to the environment and routine.

We saw a lot of negative behaviours overnight and a lot of pacing at our glass window during the day as well when they first lost that exhibit. So, we did have a lot of stereotypic behaviour to levels we were not happy with. So, we tried that carcass feed and he was good, he wasn’t pacing at the window.

(ID08)

Thus, keepers often applied enrichment practices to address behavioural problems, such as pacing, rather than to stimulate exploration, curiosity, and other natural behaviours.

… like the pumas, in the afternoon we will provide with enrichment—the male especially, walking back and forth was reduced—and when we stopped providing that often… it came back.

(ID21)

However, many keepers who attempted to implement enrichment in this manner identified the inadequacies of such practices for effectively addressing underlying causes of more complex welfare issues.

When I see the animal pace inside the window and hit his head off the window… then I would take the animal back off display and I would put different toys and treats in… but that then became conditioned where that would then happen every time. So, I didn’t fix the problem.

(ID03)

### 3.3. Struggle to Understand the Goal

Zoos often provided enrichment to address multiple goals, without delineating the intended purpose and means of selecting enrichment practices. To implement enrichment effectively, keepers attempted to fulfil regulations that were often ambiguous while navigating the diversity of goals that enrichment may be intended to deliver. Enrichment practices sometimes fulfilled one goal and impeded another goal, creating further challenges for keepers to ascertain the most appropriate enrichment to use.

#### 3.3.1. “Big Differences in Opinion”

The goals that enrichment is intended to achieve should guide the practices that keepers apply. Yet, keepers were often unsure of which goals to fulfil and thus uncertain of which practices to implement.

I didn’t quite understand the importance of enrichment, to me it was *‘oh, I just have to throw something in there, and that’s that*’… I think that’s where it is falling down, some newer keepers just aren’t learning the goal of enrichment is to replicate behaviours and to encourage these natural behaviours.

(ID34)

Keepers most often provided enrichment to fulfil animal needs. Yet, these needs can only be deduced from the behaviour of animals.

Our biggest focus is encouraging species-appropriate behaviour, looking at behaviours in the wild, what they do, and trying to give them the opportunity as much as possible, within reason, to display those behaviours.

(ID04)

In particular, keepers often utilised positive and negative behavioural indicators to ascertain whether an enrichment practice fulfilled an important need of animals. Sometimes keepers considered a practice effective only if a positive behavioural response was observed, such as increased exploration, socialisation, or play.

If it wasn’t something that the cat enjoyed or that they didn’t care about then you just don’t do it anymore because if it’s not benefitting the cat then there’s no point in doing it.

(ID17)

At other times, keepers deemed negative behavioural responses, such as fear, to be natural and thus central to enrichment.

For a snow leopard we might give them tiger bedding, which for them we have observed fear responses in snow leopards when they have seen tigers in adjoining exhibits. So that, potentially, would be a scary animal for them… it’s still eliciting a natural response.

(ID16)

Some keepers deduced more arbitrarily that any response from an animal was sufficient to suggest enrichment was serving a function. Thus, enrichment that elicited no behavioural change from an animal was likely to be removed.

Any enrichment is better [than] no enrichment, but if they don’t respond to it then I consider that bad enrichment, it’s just like throwing nothing, it’s the same as not giving anything.

(ID21)

In contrast, other keepers maintained that providing enrichment was necessary even in the absence of an observed behavioural response. Consequently, keepers were often unsure of the desired enrichment outcomes they intended to accommodate.

… a lot of people come back and say why are we giving spices they are not doing anything with them, but I don’t necessarily think that… because there are so many variables that go into these things.

(ID02)

Keeper assessments of enrichment were also largely contingent on individual interpretations of success. Thus, enrichment practices often diverged between different keepers to align with their individual expectations and interpretation of animal responses.

For the most part we would be on the same page, but not always, and I am just recalling in my head an example of a tiger responding to a keeper, and the other keeper that I was with thought it was actually a positive reaction, and I said ‘*I actually don’t think this is a positive at all, no I think this is a negative reaction*’… I was really, really surprised that we differed so much in our interpretation of her behaviour.

(ID18)

#### 3.3.2. “Hard to Explain the Rules”

Zoo managers rarely specified which outcomes enrichment was supposed to deliver as well as the reason to achieve these outcomes. In addition to goals related to animal welfare, enrichment also partially fulfilled other goals within zoos, such as adhering to accreditation criteria, improving visitor experience, and reducing veterinary intervention.

Enrichment wasn’t as much behaviour-based, it was more just—well, to be [regionally] accredited we have to have enrichment programmes and we have to have an enrichment evaluation.

(ID34)

Zoo protocols also tended to provide direction and guidance on how to achieve some, but not all, goals. Zoo managers did not always define the broader purpose or benefit of addressing distinct goals with enrichment practices.

[The boss] was purely looking at the aesthetics and what the visitor wanted to see.

(ID01)

Additionally, the criteria keepers used to select or exclude various enrichment practices were rarely defined explicitly by zoo managers, but rather implied from the restrictions the zoo imposed on specific enrichment practices.

For things like our tiger enclosures and our big cat enclosures, if we were to hang tyres up in there it looks like a tyre yard… I don’t know, it’s hard to explain the rules around it, but there’s different rules for different enclosures depending on what animals are in there.

(ID10)

Keepers sometimes attempted to fulfil multiple, and sometimes conflicting, goals concurrently. For example, keepers in some zoos applied enrichment to enhance visitor experience. This motivation often altered the timing and schedule of routines and could sometimes diminish the effectiveness of enrichment for the animals.

… even things like the training wall that each of the cats have to do, obviously it is in the cats’ interest and their stimulation for us to do daily … but it was also about the visitor experience people, they wanted to lock-in *‘ok, the tiger will be trained every day at two o’clock at the training wall, so all you people, this is your chance to a hundred percent see a tiger*’…

(ID18)

Contrastingly, keepers in other zoos avoided this potential source of conflict by applying enrichment to benefit only animals rather than visitors.

It’s not something we advertise to public and say ‘*come to us at two o’clock and you’ll see us providing enrichment’* it’s one hundred percent a service we provide to our animals, not a service we provide to our guests.

(ID12)

Thus, the intended outcomes of enrichment were not always clear to either keepers or the individuals who approve enrichment practices.

I think that is where we struggle because the people that are approving enrichment struggle to understand the goal aspect of some of these enrichment items.

(ID34)

### 3.4. Can’t Always Provide What You Should

Implementing enrichment to a wide range of animals presented certain challenges that often impeded the capacity for keepers to enrich all species effectively. Thus, keepers tended to provide some animals with less variable or complex enrichment in favour of more efficient practices with other animals.

#### 3.4.1. “Such Destructive Animals”

Keepers were not always able to invest the additional effort into enriching all animals equitably without adequate resources. To enrich a diversity of animals, zoos must contribute financial, physical, and human resources, but enrichment often demands too many resources when the animals are destructive.

We found it hard on that budget because the cats do go through the toys so quickly, so say we have a ball, they will destroy it and those balls are usually around a grand each (approximately 1000 USD) … so we don’t really have enough funding to replace the toys.

(ID16)

Thus, resource constraints often limited the variability of enrichment available for many of these more destructive animals.

Our sealions… because they are such destructive animals, their enrichment is mainly bought-in toys and then we are restricted by budget, so they probably don’t have a lot of variability…

(ID09)

Big cats, in particular, were not only considered destructive to objects but also to pose potential safety concerns for keepers, and additional measures to manage big cats safely often increased the demand on human resources, such as keeper availability. Thus, keepers were sometimes further hampered in their capacity to implement regular enrichment for big cats as compared with other species.

Big cats are potentially getting less now because you have got to have a second person with you… whereas, where we can go in without having to wait for a second person like our coatis, and fennec foxes and meerkats… [animals that are] smaller, enrichment comes easier, [they] would get more enrichment.

(ID09)

To meet the enrichment needs of big cats despite limited resources, keepers tended to apply a subset of available practices—practices that often demand greater invested effort.

… enrichment requires you to be really creative. And it is hard, and it is taxing, and I don’t consider myself a creative person…

(ID34)

Yet, practices that warranted extra effort were not always considered to be worthwhile. Keepers sometimes deliberated on this trade-off between effort and outcome to determine whether the perceived benefit to animals warranted the additional effort.

Animals don’t use enrichment like you think they should. So that is hard too, if you spend so much time developing these enrichment items and then the animals don’t even use it. Especially destructive animals, they are like ‘*well I’m just going to break this to get it out*’ instead of actually doing the puzzle.

(ID34)

Keepers were also unlikely to invest effort into enrichment that did not appear to benefit animals to the same extent as less taxing enrichment practices.

If it’s not providing a benefit or not seeming to elicit anything from them then you are wasting your time and you are wasting their time.

(ID17)

Consequently, keepers predominantly enriched big cats by incorporating practices that appeared most efficient.

We rely pretty heavily with the cats on sensory enrichment… it’s easy, it’s quick, and we have a lot of it.

(ID16)

Thus, the enrichment practices that were implemented did not necessarily reflect practices most suited to the species.

#### 3.4.2. “Some Animals Need More Enrichment”

To enrich a wide range of animals, keepers attempted to assess the relative benefits of enrichment to different species and adjust their practices accordingly. Keepers often modified enrichment practices for different animal species in response to personal observations, experiences, and beliefs. For example, beliefs regarding the capability of distinct animal species to comprehend and resolve complexity were reflected in this particular keeper’s experiences working with big cats and bears.

Cats are really tricky because they are not problem solvers, they are trickier to train than other species. Bears are like dogs, they are problem solvers… Whereas cats are very instinctual, it takes a lot longer to train a cat to do something.

(ID05)

Keepers also developed assumptions of the expected enrichment requirements of different species. Thus, implicit beliefs of keepers also determined practices, such as the frequency and types of enrichment provided to certain animals.

Some animals need more enrichment obviously than others. Some things like big cats you might be offering something novel every day, whereas maybe with a lizard it only needs to be once a month.

(ID01)

Keepers also integrated these beliefs into the selection of appropriate enrichment, often through a personal assessment of the relative benefits enrichment will provide to different species or individual animals.

We give them smells and things, but for animals like red pandas you wouldn’t be hiding their food because they just wouldn’t get it and you would just be frustrating an animal not enriching their lives.

(ID06)

To accommodate the needs of different big cats, keepers sometimes attempted to enhance the experiences of species that are difficult to accommodate by providing additional enrichment.

Especially with our tigers is how stagnant an enclosure can be… they don’t have those things, like different animals coming through, and conspecifics coming through… we are trying to put that in their space, prey animal smells.

(ID16)

Keepers often preferentially enriched animals that were temporarily experiencing inadequate conditions. For example, keepers often utilised various forms of enrichment to compensate for reduced stimulation of animals that would normally have been granted greater access to social interactions.

I don’t see why we shouldn’t give socially housed animals just as much enrichment as we would a lone animal. Unless it’s an animal that’s living solitary that would general be a social animal, in that case then I think they need huge amounts of enrichment to make up for that lack of social interaction.

(ID10)

### 3.5. Judge the Effectiveness

To implement enrichment effectively, existing practices need to be evaluated. Yet, few policies and procedures are available to evaluate the effectiveness of enrichment practices. Even tools and procedures that are designed to document enrichment practices and outcomes are not widely available. Indeed, the existing tools and procedures often preclude more nuanced and comprehensive appraisals of enrichment, such as the subjective appraisals of keepers. These subjective evaluations by keepers were often perceived as less compelling than more quantifiable measures. Consequently, enrichment assessments and decisions rarely accommodated the appraisals of keepers.

#### 3.5.1. “No Physical Record That We Evaluate With”

Enrichment programmes with record-keeping protocols could assist the planning, delivery, and assessments of practices. Yet, evaluations of enrichment were difficult to monitor without standardised procedures to document animal management practices and outcomes.

There is definitely a potential for growth in developing an enrichment programme that could cover all the aspects of enrichment on a proper scale, rather than just day-to-day, doing something on the whim.

(ID28)

Several zoos used data management tools to document a broad range of husbandry and enrichment practices. Databases were potentially useful for systematically storing information for different animals when used effectively.

Instead of re-inventing things all the time we are actually documenting it so that we know ‘*oh, we’ve actually tried that in the past*’ so that we know that we’ve gone through those steps and it’s all documented rather than just the unknown, or just relying on the brains of keepers remembering.

(ID05)

However, effective use of databases and rating systems also depended on the capability of keepers. Zoo managers sometimes offered enrichment assessment tools, but rarely facilitated training or provided guidance for keepers to incorporate enrichment assessment into their routine.

So then, there is a formula, don’t ask me, there is a formula that creates this, and essentially there is a score out of ten and if an item does not score a six or higher it needs to be evaluated if it’s actually meeting the goal of what the item is actually supposed to be meeting…

(ID34)

To manage and document enrichment delivery, departments within the zoo often customised standard approaches to suit their specific group of animals. To illustrate, some keepers of big cats utilised specialised calendars to schedule enrichment practices. Yet, these calendars did not always enable keepers to adapt and to record practices that were modified to suit certain conditions.

We do have a set enrichment calendar, but I guess we use our common sense a lot as well. If it says ice blocks today and it’s a 10-degree day, I’m probably not going to use ice blocks, but if it’s a 30-degree day, then maybe more so. I’m not going to be putting out a cardboard box or paper bags if it’s raining.

(ID18)

Furthermore, some of the record-keeping tools that accompanied enrichment schedules were not versatile enough. Often, these tools merely recorded whether enrichment had been applied and did not retain an accurate record of the practices.

We have just got a new enrichment calendar—it’s set for each day—and at the moment, we just judge if they did get enrichment that day. So even if you didn’t do what was on the list but you still gave them enrichment, it gets ticked off as they were enriched that day.

(ID16)

Indeed, many zoos did not use any enrichment assessment tools. Keepers with limited or no access to specific documenting tools sometimes conducted ad hoc, informal, and undocumented evaluations if at all.

It’s sort of like a mental record of what enrichment really works for some of our animals and what doesn’t... there is no physical record or anything like that that we evaluate with.

(ID10)

#### 3.5.2. “Harder to Quantify”

The criteria to measure enrichment success included a broad range of outcomes relating to short- and long-term changes in animal behaviour, disposition, and physical interaction with enrichment.

I guess we are just looking for those natural behaviours, rolling in something stinky, and cheek rubbing and scent marking, Flehmen responses… play behaviour is a big one.

(ID16)

Yet, zoos seldom developed criteria to guide evaluations. Consequently, the data that keepers collated were often limited and imprecise.

I find it really hard to judge the effectiveness of enrichment without knowing exactly what they are doing for twenty-four hours with the enrichment that you have provided… we might watch them for ten minutes twice a day, so twenty minutes out of twenty-four hours is not a very good sample size.

(ID02)

The data that keepers collated, including their subjective impressions, may not measure the experience of animals definitively, but represent useful indicators to assess enrichment practices. Yet, keepers were seldom granted opportunities to utilise these subjective impressions to inform decisions about animal management.

I guess it is unfortunate that they can’t see it for themselves, or at least they don’t take our opinion, or our thoughts, our experience on board.

(ID18)

The limited value that was attached to the opinions of keeper opinions impeded the capacity of zoos to ascertain which enrichment practices were effective. This limitation is especially pronounced because the sensory and cognitive consequences of enrichment are frequently hard to measure.

It’s really tricky because I guess it becomes subjective... it’s the things like the scents and the oils and the things that get the brain more active that is harder to quantify.

(ID01)

The personal values and preferences of keepers seemed to bias appraisals of whether enrichment practices were effective. Thus, combined with limited guidance on how to conduct evaluation, this individual discretion often generated inconsistency amongst keepers.

But in terms of things that are subjective, then it’s hard to argue because some people might just prefer doing one way than the other.

(ID11)

Ambiguous evaluation processes also diminished keepers’ confidence in their own decisions. Keepers often relied on informed judgements to decide which enrichment practices to maintain and which practices to omit, but keepers also doubted their choices, particularly without the support of protocols or distinct parameters on the enrichment selection process.

If it was continuously something that we didn’t see to have any benefit over a longer period of time then we would probably just not give it again… we didn’t know if that was the right decision to be honest, and again just because we don’t see them interact with it, doesn’t mean they are not interacting with it, which is the problem, especially with things where you can’t really tell whether they have interacted with it or not.

(ID17)

## 4. Discussion

Zoos often introduce changes in how they manage animals [64,65]. Yet, few studies have investigated the processes that facilitate or impede these changes to established practices, such as enrichment protocols (for examples, see [66,67]). Moreover, the effectiveness of such changes in zoos are largely undocumented.

The current study provides new insight into the processes that impede or enable effective enrichment practices for big cats. To explore enrichment implementation from the experiences and perceptions of keepers, we used an inductive qualitative approach to investigate practices across multiple zoos. Our findings are presented as five themes as defined through the words of keepers: *“let’s just be cautious”*, *“purely surviving”*, *“struggle to understand the goal”*, *“can’t always provide what you should”*, *and “judge the effectiveness”*. To further consider the significance of these findings, we discuss our themes with reference to the Moore’s Process Evaluation framework [2]. In particular, we adopt Moore’s analysis of how delivery is achieved and what is delivered in practice, using three key criteria: fidelity, dose, and reach. Each of these criteria are defined within the following sections and described in relation to the themes and subthemes—as illustrated in Table 3.

### 4.1. Enrichment Fidelity

Ambiguous procedures to manage animals and impediments to transparency can hinder the quality of enrichment practices in zoos. The fidelity of enrichment practices—that is, the degree to which the practices that keepers implement is consistent with recommended standards—is particularly hard to assess because of widespread uncertainty surrounding suitable goals and evaluation criteria [68,69].

Consequently, current enrichment practices are often haphazard, sometimes devoid of clear goals or documented records of which practices were implemented (Table 3, Subtheme 5.1). Additionally, zoo policies do not stipulate how zoos can balance the suitable enrichment of animals alongside the interests of visitors, the public, or the media (Table 3, Subtheme 3.2). Thus, constraints on transparency around enrichment protocols to the public and across the industry further limit the fidelity of enrichment practices.

Without clear guidelines on the expected outcomes that enrichment is supposed to deliver, the fidelity and quality of enrichment practices are hard to gauge (Table 3, Subtheme 3.1). Although enrichment can contribute to welfare, conservation, and education [21], zoos managers rarely clarify which, if any, objectives their enrichment programme is intended to deliver [70]. Furthermore, because of concerns around how zoos evaluate enrichment practices [31,71], such as the neglect of subjective impressions or objective metrics (Table 3, Subtheme 5.2), whether these practices achieve the behavioural, cognitive, or emotional outcomes of animals remains ambiguous [72].

Limited opportunities for keepers to compare and share enrichment insights also impedes the appraisal of enrichment fidelity. The exchange of information between zoos and zoo professionals increases the prospective sample size and improves the management of scarce animals [73,74]. Keepers afforded the opportunity to network and communicate between zoos are also more confident in improving animal husbandry techniques, including enrichment [67]. Yet, keepers are not always encouraged or able to communicate freely and transparently with other zoos (Table 3, Subtheme 1.2). Thus, keepers often experience difficulties establishing comparative insights on enrichment failures and successes because of impediments to collaboration with experts, professional colleagues, and experienced colleagues.

Zoo policies often encourage professionals to refrain from public discourse or limit transparency with society more broadly. Zoos tend to promote a positive image [75] but censor particular practices from the public [76], arguably to avoid certain unpleasing aspects of zoo work discouraging visitors from attending. Yet, this study uncovered examples of this censorship extending more broadly throughout the zoo industry (Table 3, Subtheme 1.2). Policies in zoos that perpetuate this defensive approach to potential criticisms are likely to elicit further scrutiny rather than achieve a more positive portrayal of the efforts of zoos to overcome these same concerns [77].

### 4.2. Enrichment Dose

The frequency, duration, and magnitude of enrichment that animals receive in zoos—called the enrichment dose—are sometimes inadequate. Keepers attempt to provide animals with regular enrichment, but the intended dose is often reduced by other prioritised practices and procedures (Table 3, Subtheme 4.1). In particular, zoos often assigned higher priority to practices that fulfil the physiological health and safety needs of animals rather than practices that accommodate the mental health and stimulation of animals. This inclination to prioritise physical health is evident in the way zoos often prescribe and enforce husbandry routines but not enrichment practices (Table 3, Subtheme 2.1). Keepers are more likely to direct their attention to the prevention of problems and repercussions that emanate from the physical concerns of animals and, thus, are less likely to be attuned to the benefits of enrichment (Table 3, Subtheme 2.2).

In contrast to the physiological needs of animals, the social needs, mental stimulation, and freedom to choose in animals—presented higher in the Maslow’s pyramid illustration of WAZA guidelines—are commonly depicted as desirable for improved welfare but not critical for addressing basic care principles [78]. This distinction also appears evident in the tendency of zoos to prioritise husbandry practices over enrichment practices [79].

This inclination of zoos to prioritise husbandry practices tends to encourage keepers to direct their attention to the prevention of problems and not to the positive experiences of animals. Procedures and practices intended to limit safety risks and malpractice often accentuate negative welfare indicators, such as abnormal behaviours, reducing the likelihood that keepers will recognise good welfare [80]. As identified in our study, keepers often notice and attempt to redress inadequacies in basic animal care but are less likely to facilitate positive animal experiences with enrichment practices (Table 3, Subtheme 2.2). This reactive approach of providing enrichment to address welfare concerns [32] was further revealed to be related to the underlying beliefs and values of different keepers (Table 3, Subtheme 4.2).

This disinclination to provide enrichment with the same urgency as basic care may compromise long-term improvements to animal welfare. Zoos sometimes avoid the challenges associated with a more holistic animal welfare management approach by prioritising the prevention of practices that pose a risk, particularly in the short term [79]. Our findings further demonstrate this tendency to prevent negative consequences; that is enrichment practices that pose any risk may be curtailed or withheld (Table 3, Subtheme 1.1). Accordingly, keepers may fail to apply some of the most beneficial enrichment practices.

Zoos also limit some enrichment practices to avoid other potential risks associated with public relations. Societal expectations of zoos have increased pressure on managers to modify the mission, values, and practices around animal management [65]. This concern about media attention largely prompts zoos to become more vigilant and restrained in implementing and publicising various enrichment practices (Table 3, Subtheme 1.2). Thus, a risk aversive approach not only impedes enrichment delivery, but also counters some of the benefits of engaging society in effective discourse on the mission and purpose of zoos. This vigilance, however, may be misguided: increased media attention, even because of controversial matters, tends to attract visitors, potentially diversifying the people who attend zoos and increasing the opportunity for open discourse around welfare uncertainties with the public [81].

### 4.3. Enrichment Reach

Relative to other animals, some animals, such as big cats, may be granted limited access to enrichment. Zoo policies that regulate husbandry procedures as well as the beliefs and assumptions of keepers around animal enrichment may shape how this level of access, called reach, varies across animals. To illustrate, because of their destructive tendencies (Table 3, Subtheme 4.1), most species of big cats are subject to structured routines, such as standardised operating procedures. Standardised operating procedures stipulate the timing and allocation of resources to these animals (Table 3, Subtheme 2.1). As a consequence of these constraints, keepers may tend to apply enrichment practices habitually rather than sporadically [32], and these practices may not be variable enough to stimulate the cats mentally [82]. Relative to the enrichment of other animals, such as meerkats, the enrichment of big cats—animals that benefit appreciably from diverse and mentally stimulating enrichment [18,83]—may thus be inadequate.

The beliefs and assumptions of keepers may also shape the reach of enrichment practices. To provide effective enrichment to a diversity of big cats, keepers must tailor their practices to the particular conditions and characteristics of each animal [10,84]. The personal experiences of keepers with specific animals or species, and the beliefs they generate from these experiences, can shape enrichment practices. For example, keepers may vary the complexity of enrichment practices to accommodate the perceived needs and capabilities of particular cats (Table 3, Subtheme 4.2). This finding aligns with research into several species of great apes that has revealed increased enrichment provisions for the more solitary species, with the authors suggesting perceptions around reduced social stimulation led to additional enrichment practices as compensation [85]. Thus, the beliefs of keepers both impede and facilitate the reach of enrichment to different species of big cats as well as other animals in zoos.

### 4.4. Study Limitations and Further Research

To gather a broad range of insights and investigate enrichment in a diversity of contexts, we conducted our study in multiple zoos globally. We recognised that the considerable variation across nations—such as variations in cultural values, regulations, and management practices—also generated differences in the standards of animal welfare provisions and practices across zoos [29,73,86]. Therefore, to limit this variation, and to specifically include zoos that prioritise animal welfare and enrichment, we included only the zoos accredited with regional branches of the World Association of Zoos and Aquariums (WAZA).

Another limitation is the study explored the practices, procedures, and policies within zoos that might impede the implementation of enrichment protocols rather than standards, policies, and laws that are imposed on zoos by associations, governments, and other authorities. Future research should thus explore the perceptions and attitudes of zookeepers and zoo management towards these standards, policies, and laws.

Conducting research across multiple institutes also complicates data gathering [17,69]. Zoos include an extensive range of departments, each responsible for the management of diverse groups of animals. In addition, we suspected that each department was likely to develop practices specific to the habitat and needs of different animal groups. To investigate a comparable range of enrichment practices across zoos, we limited sampling to one department within each zoo: the carnivore department. Further research should explore whether the constraints to enrichment for carnivores extend to other animals.

## 5. Conclusions

The current study provides empirical insights into conditions and perspectives that impede or facilitate the implementation of enrichment of big cats in zoos. Some of the impediments identified in this study include widespread uncertainty surrounding the objectives that enrichment practices should aim to achieve, and the evaluation approaches that keepers could apply to assess these practices. Further research into enrichment decisions and processes in zoos at an organisational level could increase clarity on the multiple objectives that enrichment is intended to deliver for animals, keepers, visitors, and the public.

Zoos and industry guidelines also tend to provide more specific criteria for practices intended to address the physical health of animals than practices that provide for animal mental health. Thus, keepers sometimes implement enrichment and direct greater attention to practices that reflect this emphasis on the physical welfare of animals.

This study further identified conditions that facilitate more effective enrichment implementation, such as greater opportunities for keepers to network and share ideas between zoos and the membership organisations and associations of keepers. Widespread communication between zoos, however, is sometimes hindered by the degree to which zoos are willing, or able, to openly share information both in the industry and with the public more broadly.

## Figures and Tables

**Table 1:** Interview scheduled questions and examples of probing questions.

Scheduled Question	Probing Questions
What are the most important aspects of animal welfare?	You are mentioning they’ve got food and shelter. Are there any other aspects of welfare that you think are important?
What do you believe to be the purpose of enrichment?	You mentioned primates. Do you think they have different requirements than big cats in terms of enrichment?
How do you decide which enrichment items to keep or remove?	So, what did that look like then? What do you mean when you say a happier cat? How do you know?
How do you develop and modify enrichment?	You were talking about the possibility of having some cognitive enrichment. Is that something you’ve tried to develop for big cats?
How do you think enrichment should be evaluated?	You are talking about records. What kind of record keeping do you use at the moment?

**Table 2:** Iterative development of themes.

Initial Codes	Initial Theme(s)	Final Theme
Time to sit and observe	Time with animals	Judge the effectiveness
We use our common sense		
Not consistent	Documenting enrichment	
More science-based		

**Table 3:** Interpretation of themes and subthemes in relation to fidelity, dose, and reach.

Themes	Subthemes	Fidelity (f), Dose (d), Reach (r)
1. Let’s just be cautious	1.1 sometimes things go wrong	d
	1.2 don’t think we really want the attention	f, d
2. Purely surviving	2.1 health and wellbeing as number one priority	d, r
	2.2 animals in zoos would still survive without enrichment	d
3. Struggle to understand the goal	3.1 big differences in opinion	f
	3.2 hard to explain the rules	f
4. Can’t always provide what you should	4.1 such destructive animals	d, r
	4.2 some animals need more enrichment	d, r
5. Judge the effectiveness	5.1 no physical record that we evaluate with	f
	5.2 harder to quantify	f

## Data Availability

The deidentified data presented in this study are available upon reasonable request.
